# Effects of Different Types of Stretching on Hypertension: A Systematic Review with Exploratory Meta-Analysis

**DOI:** 10.3390/jfmk11020164

**Published:** 2026-04-22

**Authors:** Irene-Chrysovalanto Themistocleous, Charalambos Michael, Stelios Hadjisavvas, Elena Papamichael, Michalis A. Efstathiou, Christina Michailidou, Manos Stefanakis

**Affiliations:** Physiotherapy Program, Department of Health Sciences, University of Nicosia, Nicosia 1700, Cyprus; themistocleous.i@unic.ac.cy (I.-C.T.); xaralampos.m416@gmail.com (C.M.); hadjisavvas.s@unic.ac.cy (S.H.); papamichael.el@unic.ac.cy (E.P.); efstathiou.m@unic.ac.cy (M.A.E.); michailidou.c@unic.ac.cy (C.M.)

**Keywords:** stretching, flexibility exercise, hypertension, elevated blood pressure, systolic blood pressure, diastolic blood pressure

## Abstract

**Background:** Stretching exercises are strongly recommended as part of exercise training programs; however, their effects on blood pressure (BP) and other related cardiovascular parameters in adult individuals with elevated BP (pre-hypertension) or hypertension remain unclear. **Methods:** A systematic search was conducted in PubMed and databases accessed via the EBSCO platform up to 30 September 2025, following the PRISMA guidelines. An additional search of Scopus was performed on 8 April 2026. Studies eligible for inclusion were randomized controlled trials, randomized crossover trials, non-randomized clinical trials and single-arm trials investigating stretching interventions in adults with pre-hypertension and or hypertension. Risk of bias assessment was performed using RoB 2 for randomized trials and ROBINS-I for the non-randomized trials. A random-effect meta-analysis was performed when at least two studies reported sufficiently comparable BP outcomes. The quantitative synthesis was considered exploratory. **Results:** Eleven records published between 2014 and 2025 met the eligibility criteria and were included. All protocols used static stretching, although only a small number were clearly described as active stretching. The results were heterogeneous across the design, duration of intervention and outcomes. Chronic interventions more often reported favorable changes in indices of arterial stiffness, whereas acute interventions demonstrated more variable immediate BP responses. In the exploratory meta-analysis, the pooled estimate suggested a reduction in systolic blood pressure (SBP) in favor of stretching; however, this effect did not reach statistical significance (mean difference (MD) = −5.39 mmHg, 95% confidence interval (CI): −11.32 to 0.53; I^2^ = 0%). For diastolic blood pressure (DBP), the pooled estimate favored stretching and reached statistical significance (MD = −3.93 mmHg, 95% CI: −7.25 to −0.60; I^2^ = 0%). In sensitivity analyses including a third study, the pooled effects remained in favor of stretching for systolic BP (MD = −6.6 mmHg, 95% CI: −12.2 to −1.0; I^2^ = 56%) and diastolic BP (MD = −5.4 mmHg, 95% CI: −7.1 to −3.7; I^2^ = 8%). These pooled estimates should be interpreted with caution due to the small number of studies, heterogeneity in study design and participant characteristics, and overall limitations in methodological quality. Secondary findings suggested possible improvements in selected vascular parameters, including brachial–ankle pulse wave velocity, augmentation index, and cardio–ankle vascular index, whereas acute responses were more variable and protocol-dependent. Overall, the level of evidence was limited, with most randomized trials judged as having some concerns and non-randomized studies judged as having a critical risk of bias. **Conclusions:** Stretching interventions may improve BP and selected vascular parameters in adults with pre-hypertension and hypertension and may represent a practical adjunct within the non-pharmacological management of BP. However, the current evidence is limited by methodological heterogeneity, risk of bias, and the small number of studies available for quantitative synthesis. Therefore, the pooled findings should be considered exploratory and hypothesis-generating rather than definitive. Further high-quality randomized controlled trials are required to determine the optimal type, dose, and long-term clinical relevance of stretching interventions in this population.

## 1. Introduction

Arterial hypertension (AT) still remains one of the world’s greatest health challenges and concerns as it continues to affect cardiovascular health [1]. Applying the American College of Cardiology and American Heart Association (ACC/AHA) criteria, blood pressure (BP) is classified as: (i) normal BP when the measurement of systolic blood pressure (SBP) is <120 mmHg and diastolic blood pressure (DBP) is <80 mmHg, (ii) elevated when SBP is 120–129 mmHg and DBP is <80 mmHg, (iii) stage I AT when SBP is 130–139 mmHg or DBP is 80–89 mmHg, and (iv) stage II AT when SBP is ≥140 mmHg or DBP is ≥90 mmHg [2]. Controlling AT is one of the most common reasons for physician visits and long-term medication prescription [3,4]. Definitions and classification for BP have changed over the years and differ across guideline frameworks. This should be considered when interpreting studies published under different guideline frameworks. More specifically, the 2024 European Society of Cardiology (ESC) guidelines continue to define AT ≥ 140/90 mmHg, where most individuals must be treated [5]. At the same time, these guidelines broaden the category of elevated BP to include SBP 120–139 mmHg and DBP to 70–89 mmHg [6], although medication is recommended mainly for those at higher cardiovascular risk [5]. Initial pharmacological treatment for AT includes beta-blockers, angiotensin-converting enzyme inhibitors, calcium channel blockers, diuretics and other medications [7].

The 2024 ESC guidelines for non-pharmacological treatment recommended that individuals with AT should minimize alcohol consumption, maintain or improve their weight, terminate smoking and restrict sodium [8,9]. In addition, whereas previous AT guidelines recommended regular exercise without specific details, the 2024 ESC guidelines provide more specific recommendations: at least 150 min/week of moderate intensity aerobic exercise or 75 min of vigorous intensity aerobic exercise, combined with low to moderate intensity dynamic or isometric resistance training 2–3 times/week [10]. Evidence from various studies demonstrated that exercise is an effective non-pharmacologic intervention for the prevention and management of AT [11,12,13,14,15,16,17,18]. The magnitude of BP reduction seems to depend on the specific parameters of exercise interventions, and previous studies demonstrated BP-lowering effects across different types of exercise [19]. In addition, the ACSM has provided detailed exercise recommendations for individuals with AT, including aerobic, resistance and flexibility exercises (Table 1).

Flexibility exercises may be classified based on the movement pattern (static, dynamic) and the mode of application [how the stretch is produced] (active, passive) [20,21]. In the literature, there are mixed definitions regarding active and passive stretching, often defining it based on the way it is applied. If it is applied by another individual, it is classified as passive, and if it is self-performed, it is classified as active [21]. When the position is held for a specific duration, the stretching is classified as static, and when there is repeated movement through an ROM without an end-position hold, it is dynamic [21].

Static stretching can be performed passively by another individual, or actively by the individual performing the stretch by moving the limb through its full ROM to the end ranges and repeating several times [21]. However, during active stretching, the movement involves a concentric contraction of the antagonist muscle groups to the agonist muscles that are being stretched, allowing the joint to reach and maintain its full ROM [20]. On the other hand, passive stretching uses an external force (e.g., application by an operator, gravity, or device/equipment, hands) in order to rotate the joint up to the end ROM without requiring any active effort from the individual [22]. In addition, constant-angle involves maintaining the same end ROM, leading to a progressive decline in perceived discomfort of the stretched limb [20]. During passive static stretching, an external force is applied in order to reach the maximal ROM; thus, only a slight isometric contraction of antagonist muscle groups will be performed during the application of stretching exercises. Nevertheless, studies examining the application of stretching interventions on BP often insufficiently describe the type of stretching, leading to uncertain classification and creating an important challenge for the synthesis of evidence.

Although aerobic and resistance exercise have been widely studied for BP management, some individuals may not tolerate or adhere to more demanding exercise modalities. Flexibility interventions may therefore provide a more feasible option for certain individuals. Nevertheless, their effects on BP and other related cardiovascular parameters have been examined far less extensively, leaving a gap regarding the optimal exercise parameters and clinical impact in this clinical population.

Therefore, the aim of this systematic review was to evaluate the available literature on the flexibility interventions in individuals with elevated BP and AT, to examine their effects on BP and related cardiovascular health parameters and try to identify which exercise parameters may be associated with greater improvements in BP.

## 2. Materials and Methods

The study protocol was registered in the Open Science Framework (OSF) (https://doi.org/10.17605/OSF.IO/W2FPA). This systematic review was conducted and reported in accordance with the Preferred Reporting Items for Systematic Reviews and Meta-Analyses (PRISMA) 2020 statement [23]. The PRISMA 2020 checklist is provided in Supplementary Table S1. Ethical approval was not required because this study was based on previously published data and did not involve direct patient participation.

A systematic literature search was conducted in PubMed (including MEDLINE-indexed records), Scopus, and databases accessed via the EBSCO platform (e.g., CINAHL and SPORTDiscus) from the databases’ inception to 30 September 2025. Additional studies were identified through manual screening of the reference lists of eligible articles. All retrieved records were imported into EndNote software (v2025.3.1, Clarivate Analytics, Philadelphia, PA, USA) for deduplication and management. The search strategy was based on combinations of keywords related to stretching and blood pressure, including “stretching”, “flexibility exercises”, “hypertension”, “high blood pressure”, and “pre-hypertension”. Boolean operators (AND/OR) were used to combine search terms, and the search strategy was adapted as appropriate for each database. Full electronic search strategies for all databases, including exact search strings and applied limits, are provided in Supplementary Table S2 to ensure transparency and reproducibility. Titles and abstracts of all retrieved records were screened independently by two reviewers (ICT and SH) according to the predefined eligibility criteria. Full-text articles were subsequently assessed for eligibility. Disagreements were resolved through discussion and, when necessary, consultation with a third reviewer (EP).

The risk of bias was assessed independently by two reviewers (M.A.E. and C.M.) using the Cochrane Risk of Bias 2 (RoB 2) tool for randomized controlled trials and the Risk of Bias in Non-randomized Studies of Interventions (ROBINS-I) tool for non-randomized studies [24,25]. Any disagreements were resolved by a third reviewer (I.-C.T.).

### 2.1. Eligibility Criteria

Articles were eligible for inclusion if they met the following Population, Intervention, Comparison, Outcome, Studies (PICOS) criteria: randomized controlled trials (RCTs), randomized crossover clinical trials, single-arm trials and randomized clinical trials or non-randomized controlled trials published in the English language; adults (mean ≥ 20 years) with elevated BP (SBP 120–139/DBP 70–89 mmHg) or AT (≥SBP 140/DBP 90 mmHg); flexibility exercises such as stretching (passive, active, static, dynamic) of upper and lower limbs and core, compared to other interventions or no intervention at all; and the assessed outcomes were SBP, DBP, hemodynamic status (mean arterial pressure [MAP], heart rate [HR], cardiac output [CO], systemic vascular resistance [SVR], pulse pressure [PP], total peripheral resistance [TPR]), arterial stiffness (brachial–ankle pulse wave velocity [baPWV], cardio–ankle vascular index [CAVI], augmentation index [Aix]) and muscle flexibility. The eligibility criteria are presented in Table 2.

### 2.2. Data Extraction

Three reviewers (I.-C.T., C.M. and E.P.) independently reviewed all the eligible records and determined whether they fulfilled the selection criteria. The reviewers extracted the data into a standardized Excel spreadsheet. The information extracted from the included records was related to participant characteristics, descriptions of interventions, mean and standard deviation (SD) of outcome measures, and mean differences before and after the training period. In cases where studies used different parameters (such as baPWV, CAVI, or Aix), the outcomes were synthesized narratively.

### 2.3. Classification of Stretching Interventions

To reduce classification bias, stretching interventions were classified using specific criteria. The classification was performed independently by two reviewers (I.-C.T. & S.H.). Stretching interventions were classified as active when the end-range position was attained and maintained mainly through voluntary muscular contraction and passive when the position was maintained mainly by external force (such as gravity, bodyweight, assistance from someone else or self-application using the hands or equipment). In addition, static was defined as holding the position for a specific time, whereas dynamic stretching was defined as repeated movement through an ROM without a maintained end-position hold. Finally, when the included studies did not give sufficient details in order to determine the type of stretching, the stretching was classified as unclear. The stretching modalities were classified based on published definitions from the stretching literature and expert consensus statements [20,26].

### 2.4. Assessment of Study Quality

Two reviewers (M.A.E. and C.M.) independently assessed the risk of bias of the included studies. The Cochrane Risk of Bias 2 (RoB 2) tool was applied to randomized trials, whereas the Risk of Bias In Non-randomized Studies of Interventions (ROBINS-I) tool was applied to non-randomized studies [24,25]. Any disagreements were resolved through discussion with a third reviewer (I.-C.T.). RoB 2 assesses bias across the following domains: bias arising from the randomization process, bias due to deviations from intended interventions, bias due to missing outcome data, bias in measurement of the outcome, and bias in selection of the reported result, leading to an overall risk-of-bias judgment [27]. The judgments are classified as low risk of bias, some concerns, or high risk of bias. ROBINS-I assesses bias across seven domains: bias due to confounding, bias in selection of participants into the study, bias in classification of interventions, bias due to deviations from intended interventions, bias due to missing data, bias in measurement of outcomes, and bias in selection of the reported result [24]. The overall judgments are classified as low, moderate, serious, or critical risk of bias. The Risk-of-bias VISualization (robvis) tool was used to illustrate the results of the assessments [28].

### 2.5. Statistical Analysis

Analyses were carried out using SPSS (version 29, IBM Corp., Armonk, NY, USA). Continuous outcomes were analyzed as mean differences (MDs) with 95% confidence intervals (CIs) when the same measurement scale was used across studies. Standardized mean differences (SMDs) with 95% CIs were planned when outcomes were assessed using different scales or indices; however, because of limited comparability across studies, this approach was not widely applicable.

The main outcomes of interest were systolic blood pressure (SBP) and diastolic blood pressure (DBP), while secondary outcomes included mean arterial pressure (MAP), heart rate (HR), arterial stiffness indices, and flexibility-related outcomes. For each study, extracted data included sample size and either post-intervention means and standard deviations (SDs) or change-from-baseline values when available. To ensure consistency in the analysis of continuous outcomes, change-from-baseline values were preferred when available, as they better reflect within-group changes over time. When both post-intervention values and change scores were reported, change-from-baseline values were used where sufficient information was available. When change-score data or their associated variance measures were unavailable or insufficiently reported, post-intervention values were used, in accordance with Cochrane Handbook recommendations. Mean differences (MDs) were calculated as the difference between the intervention and comparator groups. When standard errors (SEs) were reported, these were converted to standard deviations (SDs) using the formula SD = SE × √n, where n represents the sample size. Where necessary, standard methods were used to derive missing variance measures based on the available data.

Because clinical and methodological heterogeneity was expected across studies, including differences in stretching protocols, intervention duration, comparator groups, study design, and outcome assessment methods, a random-effects model was used for the meta-analysis. Statistical heterogeneity was assessed using Cochran’s Q test and quantified with the I^2^ statistic. I^2^ values of 25%, 50%, and 75% were considered to indicate low, moderate, and high heterogeneity, respectively. A *p*-value < 0.05 was considered statistically significant. Meta-analysis was performed only when at least two studies were considered sufficiently comparable in terms of population, intervention, comparator, study design, and outcome reporting. Outcomes were not pooled when substantial methodological heterogeneity was present, when outcome metrics were not directly comparable, or when the available data were insufficient for valid quantitative synthesis.

For this reason, vascular outcomes such as brachial–ankle pulse wave velocity (baPWV), cardio–ankle vascular index (CAVI), augmentation index (AIx), and carotid–femoral pulse wave velocity (cfPWV) were synthesized narratively when pooling was not considered appropriate.

Randomized crossover and single-arm studies were eligible for inclusion in the systematic review; however, they were included in the quantitative synthesis only when sufficient information on paired differences (e.g., within-subject standard deviations or correlation coefficients) was available. When such information was not reported, crossover studies were not pooled to avoid unit-of-analysis errors and were instead synthesized narratively. Acute crossover studies were therefore not included in the meta-analysis due to insufficient reporting of paired-data variance. Given the limited number of included studies and the methodological heterogeneity across study designs and interventions, the quantitative synthesis was considered exploratory and was interpreted alongside the narrative synthesis of this review. No imputation of correlation coefficients for change scores was performed due to the limited data and exploratory nature of the analysis.

## 3. Results

### 3.1. Study Selection

**Figure 1:**
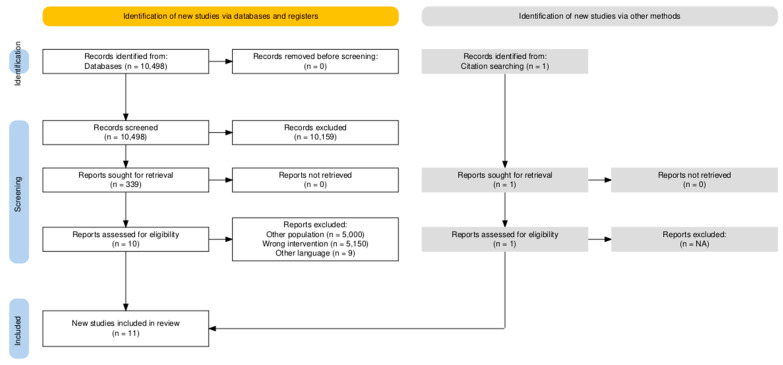
Prisma Flow chart. Note: The additional search in Scopus yielded 417 records (included in the flowchart); however, no new studies were identified after removal of duplicates.

A systematic search was conducted up to 30 September 2025 in PubMed (including MEDLINE-indexed records), Scopus, and databases accessed via the EBSCO platform (e.g., CINAHL and SPORTDiscus). A total of 10,081 records were identified through original database searching. An additional search of Scopus was conducted on 8 April 2026, in order to ensure the comprehensiveness of the search. This new search yielded 417 records; however, no new records were identified after duplicate removal. Therefore, a total of 10,498 records were identified and screened, as no records were removed before the screening process. Of these, 10,159 records were excluded during the screening process based on title and abstract, leaving 339 records for full assessment. After full-text review, 10 studies met the PICOS criteria (Table 2) and 329 records were excluded. One additional record was identified through manual screening of reference lists. In total, 11 studies met the eligibility criteria and were included in this review, as illustrated in the PRISMA flow diagram (Figure 1).

### 3.2. Study and Participant Characteristics

The characteristics of the included studies are presented in Table 3. More detailed numerical results for the included studies are presented in Supplementary Table S3. The included studies were published between 2014 and 2025. Five studies were randomized controlled trials (RCTs) [29,30,31,32,33], one study was a non-randomized controlled trial [34], three studies were randomized crossover trials [35,36,37], and two studies were single-arm trials [38,39]. Sample sizes ranged from 10 to 98 participants and mean age ranged from 22 to 72 years. Some studies included both male and female participants [29,30,37,38,39], whereas the remaining studies included only male [34,35] or only female participants [31,32,33,36]. Ten studies included participants with elevated BP (pre-hypertension) [29,30,31,32,34,35,36,37,38,39], whereas one study included participants with AT [33].

### 3.3. Intervention Characteristics

The included interventions involved stretching exercises targeting both the upper and lower body [29,31,32,34,36,38], the lower body only [30,33], the trunk only [37], or the upper back/neck/shoulder region [39]. In some studies, interventions involved stretching both upper and lower limbs and also included the neck and trunk [29,32,34,38]. Comparator groups received either no exercise intervention [31,32,33,34], walking [29], or foam rolling [30]. In the crossover trials, comparator conditions included resistance exercise alone or in combination with stretching [35], as well as vibration foam rolling (VFR) or non-vibration foam rolling [36]. Two studies were single-arm trials and therefore did not have a comparator group [38,39].

All included studies used static stretching. Among these, West et al. (2024) [37] clearly described passive stretching, whereas Mevada et al. (2024) [30] and Reyes et al. (2025) [39] described active stretching. In addition, Wong and Figueroa (2014) [31] included both active and passive stretches, whereas Silva et al. (2019) [35] described at least part of the protocol as passive. The remaining studies did not explicitly specify whether the stretching was passive or active and were therefore classified as unclear with regard to stretching modality rather than categorized by assumption.

Intervention frequency ranged from acute protocols to repeated weekly programs. Specifically, two studies used one acute session [30,39], one study involved two visits [37], two studies used three acute sessions [35,36], one study used three sessions per week [31], and five studies used five sessions per week [29,32,33,34,38]. Total intervention duration ranged from 4 weeks [33,34] to 6 weeks [32], 8 weeks [29,31], and 6 months [38].

Reported outcomes included systolic blood pressure (SBP) [29,30,33,34,35,36,37,38,39], diastolic blood pressure (DBP) [29,30,31,33,34,35,36,37,38], aortic pulse wave velocity (aortic PWV) [31], aortic SBP (aSBP) [31], aortic DBP (aDBP) [31], augmentation index (AIx) [31], heart rate (HR) [30,31,33,34,35,36,39], AIx adjusted to 75 beats·min−1 (AIx@75) [31], augmented pressure (AP) [31], brachial–ankle pulse wave velocity (baPWV) [31,32,33,34], femoral–ankle pulse wave velocity (faPWV) [31,33], mean arterial pressure (MAP) [29,31,34,36,39], pulse pressure (PP) [34,36], handgrip strength [34], arm strength [36], flexibility [34,36,38], low-frequency component of SBP (LFSBP) [31], cardio–ankle vascular index (CAVI) [34,38], reactive hyperemia index (RHI) [38], ankle brachial pressure index (ABI) [38], ankle BP [32,33], Rate Pressure Product (RPP) [30,35], oxygen saturation (SpO2) [35], root mean square of successive differences (RMSSD) [35], central BP [37], and carotid–femoral pulse wave velocity (cfPWV) [33,37].

### 3.4. Methodological Quality of Included Studies

**Figure 2:**
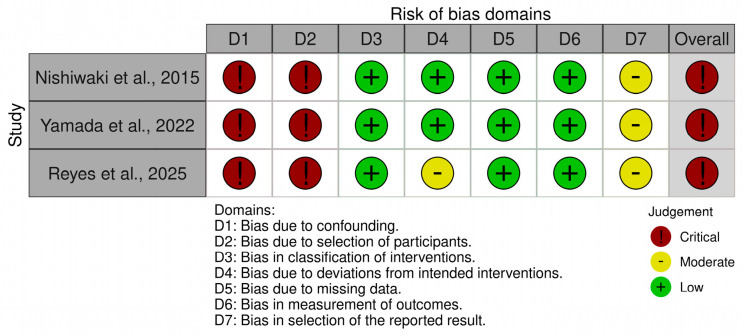
Summary of the ROBINS-I assessment for non-randomized studies [34,38,39]. ROBINS-1: Risk of Bias Assessment tool for Non-randomized Studies. Judgments were classified as low, moderate, or critical risk of bias.

**Figure 3:**
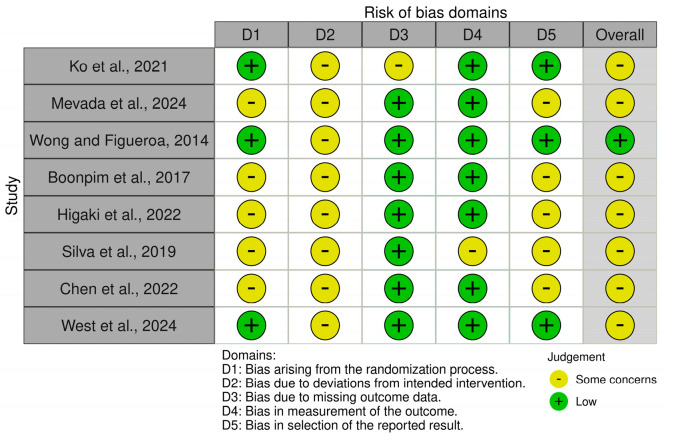
Summary of the RoB 2 assessment for randomized trials [29,30,31,32,33,35,36,37]. RoB 2: Revised Cochrane risk-of-bias tool for randomized trials. Judgments were classified as low risk, some concerns, or high risk of bias.

Figure 2 summarizes the ROBINS-I assessment for the non-randomized studies, whereas Figure 3 summarizes the RoB 2 assessment for the randomized trials. The majority of the included studies (*n* = 8) were randomized trials [29,30,31,32,33,35,36,37] and were assessed using RoB 2 [27], whereas three non-randomized studies [34,38,39] were assessed using ROBINS-I [25].

Overall, the methodological quality of the included evidence was limited. Most randomized trials were scored as having some concerns, and only one randomized study (Wong and Figueroa, 2014 [31]) was scored as low risk of bias. The most common concerns among randomized trials were related to insufficient reporting of the randomization process and allocation concealment, as well as the lack of blinding, which is inherently difficult to achieve in exercise-based interventions [29,30,31,32,33,36,37].

All non-randomized studies were judged as having a critical risk of bias, mainly due to confounding, participant selection methods, the absence of randomization, and the use of single-arm or non-randomized designs [34,38,39]. In particular, the single-arm design used by Yamada et al. [38] and Reyes et al. [39], as well as the non-randomized allocation applied by Nishiwaki et al. [34], increased the risk of selection bias and reduced internal validity.

Across all included studies, the most common methodological limitations were small sample sizes, incomplete reporting of methodological procedures, and limited blinding. Nevertheless, outcome assessment was generally based on standardized blood pressure and vascular measures, which may have reduced the likelihood of substantial measurement bias [29,31,32,33,34,36,37,38]. Therefore, the overall findings of this review, including the pooled estimates from the exploratory meta-analysis, should be interpreted with caution.

### 3.5. Effects of Stretching on Blood Pressure Parameters

#### 3.5.1. Systolic Blood Pressure (SBP) and Diastolic Blood Pressure (DBP) Findings

Most studies indicated that stretching exercises may be beneficial for BP outcomes. Specifically, favorable effects were reported for peripheral SBP [29,30,31,36,37,39], aortic SBP [31], central SBP [37], ankle SBP [32], central DBP [37], and peripheral DBP [29,30,37]. On the other hand, some longer-term studies did not report improvements in BP parameters following stretching exercises [34,38]. In most studies, the interventions used mainly static stretching, targeting both upper and lower limbs [29,31,32], lower limbs [30], trunk [37], and upper limbs [39]. These stretching protocols were compared with either a non-active control group or an active control group. Relative to the non-active control group, stretching groups were associated with significant improvements in BP parameters [31,32,37]. In addition, favorable findings were also reported when the stretching group was compared with the active control group, such as foam rolling (FR) [30] or walking [29]. In addition, combining stretching with FR produced greater improvements than stretching alone [36], whereas resistance training (RT) demonstrated greater improvements in BP parameters than stretching alone [35].

#### 3.5.2. Mean Arterial Pressure (MAP) and Rate Pressure Product (RPP)

Stretching interventions were also associated with significant improvements in MAP following stretching targeting upper limbs only or both the upper and lower limbs [29,31,39] compared with an active control group [29], a non-active control group [31], or in a single-arm trial without a comparator group [39]. In contrast, the rest of the studies showed no changes in MAP following stretching alone or combined interventions [34,36]. Findings for RPP were mixed: one study reported no significant changes following stretching exercise [30], whereas another reported higher values when stretching was combined with RT [35].

#### 3.5.3. Vital Signs and Root Mean Square of the Successive Differences (RMSSD) Findings

The majority of the studies reported no significant reductions in heart rate (HR) following stretching exercises [30,31,33,34,36]. On the other hand, one study observed increases in HR when stretching was performed before RT [35], and another reported increased HR after applying shoulder and neck stretching [39]. These findings may indicate that HR responses depend on the specific protocol and its physiological mechanism of action. Additionally, saturation (SPO2) was reduced more following stretching exercises when compared with other active interventions [35]. Similarly, RMSSD was significantly reduced when stretching was combined with RT [35].

#### 3.5.4. Vascular Endothelial Function/Arteriosclerosis Index Findings

Findings for brachial–ankle PWV (baPWV) were mixed. Two studies found no significant change [31,33], whereas the rest found significant improvement [32,34] following the intervention. In addition, similar mixed results were found for femoral–ankle PWV (faPWV) and carotid–femoral PWV (cfPWV). One study reported significant improvement in faPWV [33], whereas another found no significant improvement [31]. For cfPWV, one study found no significant change [33], whereas another found lower cfPWV values post intervention; however, the difference was no longer significant after adjustment for changes in MAP [33]. No significant changes were found for aortic pulse wave velocity (PWV) [31]. Other vascular endothelial function and arteriosclerosis indices, such as augmentation index (Aix) and Aix adjusted to 75 beats min^−1^ (Aix@75AP), low-frequency component of SBP (LFSBP), and cardio–ankle vascular index (CAVI), were improved significantly post-exercise intervention [31,34]. On the other hand, one study had different results, reporting no significant changes for CAVI, reactive hyperemia index (RHI) [38] or ankle brachial pressure index (ABI) [38].

#### 3.5.5. Flexibility and Strength

Flexibility improved significantly following stretching exercises for upper and lower limbs [34,38], and when combined with VFR [36], it had greater improvements than applying stretching alone or combined with FR [36]. In addition, handgrip strength and arm strength improved following stretching targeting the upper and lower limbs together with the trunk [34], as well as after combined interventions [36].

Because the included studies differed in duration of intervention and design, the findings were synthesized separately according to acute vs. chronic effects and randomized vs. non-randomized trials.

### 3.6. Acute and Chronic Effects of Stretching

Acute stretching studies [30,35,36,37,39] examined the cardiovascular responses following one to three single stretching sessions or maneuvers. Overall, the results were heterogeneous and protocol-dependent (population, exercise protocol, outcome). The acute studies demonstrated mixed but sometimes favorable hemodynamic responses. More specifically, Mevada et al. (2024) [30] and West et al. (2024) [37] reported reductions in BP following the stretching protocol, whereas Reyes et al. (2025) [39] observed a brief reduction in SBP during the execution of the maneuver. On the other hand, Chen et al. (2022) [36] and Silva et al. (2019) [35] demonstrated mixed acute hemodynamic responses, with no clear evidence of a stable post-intervention reduction in BP.

On the other hand, six studies [29,31,32,34,38] examined the chronic effects of stretching and more often found favorable effects on vascular stiffness parameters than on hemodynamic outcomes. Nevertheless, Ko et al. (2021) [29] and Wong & Figueroa (2014) [31] additionally stated favorable effects on BP following long-term stretching. However, Yamada et al. (2022) [38] did not demonstrate improvements in either BP or vascular stiffness parameters. Overall, the chronic evidence suggests that repeated stretching over a period from several weeks to months may be more consistently linked with vascular improvements than with lowering BP.

### 3.7. Randomized and Non-Randomized Studies

Randomized studies [29,30,31,32,33,35,36,37] provided the strongest evidence because their design allows for better control of bias, although this does not necessarily imply better effects. Among these studies, four studies examined the chronic effects of stretching [29,31,32,33] and four examined the acute effects [30,35,36,37]. In addition, [35]. Nishiwaki et al. (2015) [34] was a non-randomized chronic study and the rest of the studies were two single-arm studies [38,39], chronic studies and acute studies. Overall, chronic randomized studies often supported improvements in arterial stiffness parameters and, in some studies, reductions in BP parameters. On the other hand, acute studies demonstrated immediate BP responses. Non-randomized and single-arm studies provided additional but more exploratory evidence ranging from arterial stiffness improvement without BP reduction [34] to no chronic benefits [38] and a brief acute SBP reduction [39].

### 3.8. Exploratory Meta-Analysis Results

Exploratory meta-analysis was feasible only for a small number of non-acute controlled studies that reported sufficiently comparable brachial or peripheral blood pressure outcomes. A sensitivity analysis excluding studies at higher risk of bias was considered. However, given the very small number of studies available for pooling, such an analysis was not feasible because it would have resulted in insufficient data for meaningful quantitative synthesis.

**Figure 4:**
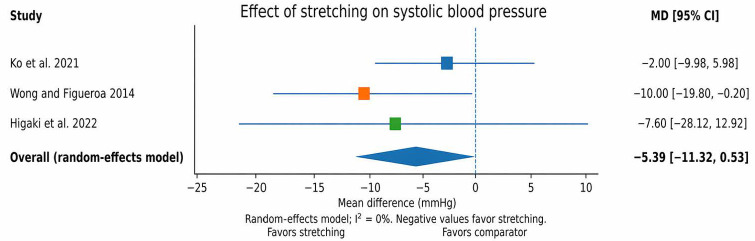
Forest plot for systolic blood pressure (post-intervention means). Negative values favor stretching [29,31,33].

**Figure 5:**
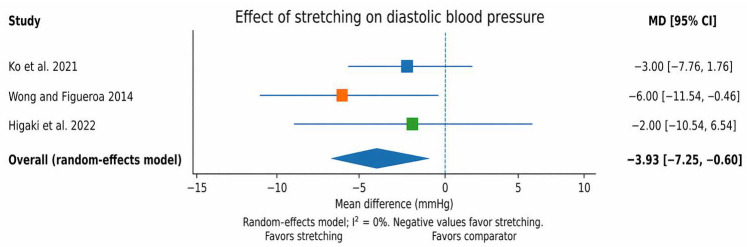
Forest plot for diastolic blood pressure (post-intervention means). Negative values favor stretching [29,31,33].

The pooled effect was estimated as a mean difference (MD) in mmHg using a random-effects model. The pooled estimate suggested a reduction in systolic blood pressure (SBP) in favor of stretching; however, the result was not statistically significant (MD = −5.39 mmHg, 95% CI −11.32 to 0.53; I^2^ = 0%). For diastolic blood pressure (DBP), the pooled estimate favored stretching and reached statistical significance (MD = −3.93 mmHg, 95% CI −7.25 to −0.60; I^2^ = 0%). These findings should be interpreted cautiously, given the small number of pooled studies and the exploratory nature of the quantitative synthesis. The pooled effect for SBP is presented in Figure 4, whereas the pooled effect for DBP is presented in Figure 5.

**Figure note:** These plots include the non-acute randomized controlled trials with sufficiently comparable brachial/peripheral blood pressure outcomes: Ko et al. [29], Wong and Figueroa [31] and Higaki et al. [33]. Wong and Figueroa reported [31] means as mean ± SE; these SE values were converted to SD for plotting. Because several other studies used acute, crossover, single-arm, or non-randomized designs, they were not pooled in these figures.

Acute studies were not pooled because of substantial methodological heterogeneity, crossover or within-subject designs, and insufficient reporting of paired-data variance. More specifically, Mevada et al. [30] reported lower post-intervention SBP and DBP after active stretching compared with FR, whereas West et al. [37] demonstrated acute reductions in peripheral BP after trunk stretching compared with seated control. In contrast, Chen et al. [36] found that static stretching alone increased SBP, while adding non-vibration FR reduced this response. Therefore, the acute evidence was synthesized narratively [30,36,37]. Furthermore, Nishiwaki et al. [34] and Yamada et al. [38] were not included in the quantitative synthesis because of their non-randomized or single-arm designs. Boonpim et al. [32], Silva et al. [35], and Reyes et al. [39] were also not pooled because their outcomes or protocols were not sufficiently comparable with those of the other controlled trials [32,34,35,38,39]. Publication bias was not formally assessed because fewer than 10 studies were available for each pooled analysis.

## 4. Discussion

The present systematic review with exploratory meta-analysis evaluated the effects of stretching exercises on BP and related cardiovascular parameters in adults with elevated BP or AT. The main findings suggest that stretching interventions may have favorable effects on BP and selected vascular stiffness parameters in this clinical population; however, these findings should be interpreted with caution, given the limited evidence base.

Clearer evidence was observed when the findings were separated based on stretching protocol duration and study design, because randomized trials represented the more methodologically robust part of the evidence and helped form the main part of the interpretation. Nevertheless, most randomized trials were scored as having some concerns, whereas all non-randomized studies were judged as having a critical risk of bias. These studies suggested that stretching interventions may be associated with favorable effects on vascular health and, in some cases, BP parameters; however, the magnitude and consistency of these effects varied across protocols and outcomes. On the other hand, while single-arm and non-randomized studies had a higher-risk of bias, they contributed with supportive and exploratory evidence regarding possible physiological responses and longer-term changes. Formal stratified analyses according to stretching modality were not feasible because only a small number of studies explicitly specified whether stretching was active or passive, whereas most protocols provided insufficient detail for reliable classification.

In addition, acute studies demonstrated mixed immediate hemodynamic responses, with some reporting brief reductions in BP parameters, while other studies demonstrated more variable and/or stretching-dependent effects. In contrast, chronic interventions more consistently suggested improvements in vascular stiffness parameters, with less favorable responses in BP parameters. Overall, stretching interventions may have a role in cardiovascular health; however, the magnitude, consistency, and clinical relevance of these effects remain uncertain because of heterogeneity in study design, intervention characteristics, and assessed outcomes. This heterogeneity is physiologically meaningful, as acute and chronic protocols, as well as active and passive stretching modalities, may induce distinct cardiovascular and vascular responses.

Although these findings suggest that stretching exercise may represent a useful adjunct within non-pharmacological BP management, especially for those who may not tolerate or adhere to more demanding exercise modalities and interventions, they should be interpreted with caution because of the small number of pooled studies, the heterogeneity of the participants and exercise protocols, and concerns regarding the risk of bias. Only a very small number of studies were sufficiently comparable for quantitative synthesis, and although the exploratory analysis suggested lower SBP and DBP in favor of stretching, the small evidence base substantially limits the confidence, robustness, and generalizability of these findings. Therefore, the pooled estimates should be considered exploratory and hypothesis-generating rather than definitive. Accordingly, the pooled results should not be interpreted as reflecting a uniform effect of all stretching interventions, as combining heterogeneous protocols may obscure potentially important differential effects.

Although the number of pooled studies was small, the observed reductions in resting SBP and DBP may still be clinically relevant; however, this interpretation remains tentative given the limited and heterogeneous evidence base [13,14,18]. Therefore, flexibility-based exercises should not necessarily be considered only in relation to mobility, although their potential cardiovascular effects require confirmation in larger and more methodologically robust studies.

The comparison between stretching and other interventions warrants attention. The study by Ko et al. [29] found that stretching was associated with greater improvements in sitting SBP and other BP indices post-stretching intervention than post-brisk walking. Nevertheless, as these findings come from a single study, they should be interpreted with caution and not be generalized. In addition, Wong and Figueroa [31] demonstrated significant reductions in brachial and aortic BP parameters after 8 weeks of stretching in obese postmenopausal women. These findings are noteworthy because walking is already being established as an effective exercise modality for BP management [17,18]. Several mechanisms have been described in the literature that may explain the effects of stretching on BP. The physiological mechanisms of stretching-related cardiovascular responses should be considered separately for acute versus chronic interventions and, where possible, for active versus passive stretching, as these approaches may involve different autonomic, circulatory, and vascular mechanisms. However, these findings are based on a very limited number of studies and are not sufficient to support firm conclusions regarding the superiority of stretching over established exercise modalities.

Regarding the acute effects of stretching, there is a tendency for a small rise in BP affecting both SBP and DBP because of short-term autonomic and circulatory responses, such as the brief compression of vessels, mechanoreflex activation and baroreflex-related changes. More specifically, during stretching, there is a brief compression of vessels, which reduces the blood flow and increases vascular resistance. As the cardiovascular system tries to maintain perfusion, this may lead to brief fluctuations in BP. This could explain the acute hemodynamic responses observed during or immediately after stretching [40]. Additionally, stretching can stimulate sensory receptors in tendons and muscles, sending signals to the nervous system that increase sympathetic outflow and produce changes in BP and HR [40]. Once stretching is discontinued, BP and HR would be expected to return toward initial levels because the compression of vessels is released and blood flow may return to the stretched area, and the autonomic balance changes and parasympathetic activity may increase [40]. This may help explain why some studies demonstrated brief reductions post-intervention. The direction and magnitude of these acute responses may depend on several protocol-specific factors, including the anatomical region stretched, the amount of muscle mass involved, whether the stretch was active or passive, stretch duration, and whether stretching was performed alone or combined with another modality such as resistance exercise or foam rolling.

On the other hand, the long-term (chronic) effects of stretching create a series of events such as the compression of vessels, the release of the compression and the blood flow restoration. This repeated cycle may increase shear stress on the vessel wall and may contribute to endothelial adaptations, improved nitric oxide bioavailability, and better vascular function, and this may reduce stiffness, showing up as lower baPWV and cfPWV or other vascular stiffness parameters [40,41].

Previous evidence [42,43,44] has also suggested that stretching interventions can improve arterial stiffness and endothelial function, and as mentioned above, these mechanisms may explain the reductions in resting BP observed after repeated exercise. The arterial stiffness findings of the present review further support this interpretation, although the evidence was less consistent than for BP. Improvements were reported for ankle SBP, baPWV, faPWV, AIx, AIx@75, LFSBP, and CAVI in several studies [31,32,33], suggesting that stretching may have beneficial vascular effects beyond conventional brachial BP. These indices should not be interpreted as interchangeable, as they reflect different physiological aspects of vascular function and arterial stiffness and differ in their measurement properties. However, these findings were not consistent across all trials, as some studies showed no significant changes in baPWV, cfPWV, aortic PWV, RHI, or ABI [31,33,38]. More specifically, pulse wave velocity-derived indices (e.g., baPWV, faPWV, cfPWV) mainly reflect arterial stiffness across different vascular territories, whereas indices such as AIx are more closely related to wave reflection and arterial tone, and CAVI reflects a broader stiffness construct that is less directly comparable with conventional pulse wave velocity measures. This variability is likely related to substantial heterogeneity in participant characteristics, baseline BP levels, medication, intervention duration, the anatomical region stretched, and the assessed vascular indices. It is also possible that vascular outcomes are more sensitive than brachial BP to differences in intervention dose and population characteristics. In support of this, a previous meta-analysis [45] reported that stretching significantly improved arterial stiffness, endothelial function and DBP in middle-aged and older adults. These findings suggest that vascular adaptations may represent one of the possible pathways through which stretching exerts cardiovascular effects. Experimental evidence has shown that stretching can acutely influence arterial stiffness and endothelial responses, but such effects may depend on the type and repetition of the stretch stimulus [46].

The mixed findings for secondary hemodynamic outcomes also merit consideration. MAP generally improved when stretching was performed as a long-term intervention [29,31,39], which is consistent with the overall reductions observed in SBP and DBP. In contrast, HR remained unchanged in most studies [30,31,33,34,36], suggesting that the antihypertensive effect of stretching may be driven more by peripheral vascular adaptations than by reductions in resting HR. The findings from previous research appear to be inconsistent because of HR discrepancies, mainly due to the exercise protocol.

The evidence for RPP, SPO2, and RMSSD was limited and inconsistent [30,35]. The study by Silva et al. [35] is particularly important because it indicates that when stretching is combined with resistance exercise, the cardiovascular response may reflect the integrated effect of both components rather than stretching alone. Accordingly, stretching performed before or after resistance exercise should not be assumed to have the same hemodynamic profile as isolated stretching. This is paramount because RT increases the cardiac workload as the amount of active muscle mass increases, considering previous studies on stretching reporting that it can produce higher RPP values [47]. Future studies should compare the cardiac load during these stretching protocols, especially due to the multiple comorbidities this clinical population has. Another relevant finding of this review is that stretching consistently improved flexibility [34,36,38], which is expected given the nature of the intervention but may also have clinical significance. Improved flexibility may facilitate adherence to broader exercise participation, reduce movement discomfort and support functional capacity in middle-aged and older adults. This may be particularly important in individuals with AT, obesity, low exercise tolerance, or musculoskeletal limitations for whom aerobic training may be more demanding. In this sense, stretching may serve both as an intervention with modest antihypertensive potential and as a gateway modality that encourages engagement in a more comprehensive exercise program.

The discrepant findings across studies likely reflect differences in intervention design and population characteristics. For example, positive long-term effects were mainly observed in studies using repeated static stretching over 4–8 weeks [29,31,32,33,34], whereas the 6-month single-arm study by Yamada et al. [38] showed no significant BP changes despite improved flexibility. This may be partly explained by the small sample size of participants, the absence of a control group, the usage of antihypertensive medication and the relatively well-controlled baseline BP levels of the participants. Likewise, Nishiwaki et al. [34] observed improvements in arterial stiffness without corresponding brachial BP changes, suggesting that vascular adaptation may precede or occur independently of detectable reductions in BP. Therefore, a lack of change in brachial SBP or DBP should not necessarily be interpreted as an absence of physiological benefit.

From a clinical perspective, the present findings suggest that stretching may be used as a practical adjunct to current lifestyle management strategies for elevated BP and/or AT. However, the current evidence remains insufficient to support stretching as a stand-alone replacement for established exercise prescriptions. Current exercise recommendations for BP control emphasize aerobic [48,49,50] and resistance exercise [51,52,53] or both concurrently [54], and the present review does not challenge their central role. However, the available evidence indicates that stretching may also contribute to BP reduction, particularly in individuals with high-normal BP or stage I AT [29,31]. Given its low cost, minimal equipment requirements and broad accessibility, stretching exercises may be particularly useful for older adults, sedentary individuals or those with limitations to exercise. At the same time, the current evidence remains insufficient to support stretching as a stand-alone replacement for established exercise prescriptions. Nevertheless, taking together the results of this review, although some patterns were observed across studies, the current evidence is insufficient to support strong recommendations regarding the superiority of active versus passive stretching or the optimal stretching protocol for acute BP reduction versus long-term vascular benefit.

The present review has several limitations that should be acknowledged. First, the total number of included studies was small and only a limited number were eligible for exploratory quantitative synthesis. Second, considerable heterogeneity was present across studies in terms of design, stretching modality, intervention dose, comparator, and outcome assessment, which limited the interpretability, validity, and clinical applicability of the findings. Third, several studies had small sample sizes and important methodological limitations, and the overall level of evidence was limited, with most randomized trials classified as having some concerns and all non-randomized studies classified as having a critical risk of bias [25,27]. Fourth, some studies included participants with elevated BP, whereas others included patients with diagnosed AT or medicated individuals, which limits direct comparability. Fifth, the classification of stretching modality (active or passive) was often not possible because many studies did not provide sufficient procedural detail; consequently, most protocols could not be reliably categorized for subgroup interpretation. Finally, acute and long-term stretching studies were necessarily synthesized separately as they have different physiological processes and publication bias could not be formally assessed because too few studies were available for each meta-analysis. These issues mean that the pooled estimates should be interpreted with caution. Future research should prioritize adequately powered randomized controlled trials with standardized BP assessment, clearer reporting of stretching intensity and volume, passive or active application, and more detailed characterization of participant medication use and baseline cardiovascular risk. It would also be valuable to determine whether specific stretching characteristics, such as the anatomical region targeted, static versus dynamic application, session frequency, or total weekly volume, are associated with greater antihypertensive effects. In addition, future studies should compare stretching directly with guideline-recommended exercise modalities and examine whether combining stretching with aerobic or resistance exercise leads to additive clinical benefits.

## 5. Conclusions

In conclusion, stretching-based interventions demonstrated beneficial effects on BP and selected vascular outcomes in adults with elevated BP or AT. Long-term (chronic) training interventions were associated with consistent benefits in vascular stiffness indices, whereas acute interventions may induce brief responses, which seem to be protocol-dependent. Nevertheless, given the limited number of available studies and the methodological heterogeneity across trials and unclear details regarding the types of stretching protocols, these findings should be interpreted cautiously. Firm conclusions cannot yet be drawn regarding the magnitude and clinical relevance of these effects, but the available evidence of this review supports the potential role of stretching as a practical aid within non-pharmacological management. Further high-quality randomized controlled studies are required to establish the optimal type, dose, and clinical applicability of stretching interventions in this clinical population.

## Figures and Tables

**Table 1:** ACSM exercise recommendations for individuals with AT.

Component	Aerobic	Resistance	Flexibility (Stretching)
Frequency	Preferably most days of the week	2–3 sessions/week, typically with rest days between sessions for the same muscle groups	At least 2–3 sessions/week
Intensity	Moderate to vigorous, e.g., ~40–80% VO_2_R or HRR; RPE ~12–16 (6–20 scale)	Typically moderate, ~60–70% 1RM (may progress toward ~80% 1RM); beginners/older adults may start ~40–50% 1RM)	Low to moderate, stretch to the point of tightness or mild discomfort (avoid pain)
Volume/time	~20–30 min/day (continuous or accumulated bouts), aiming for ~90–150+ min/week	8–10 exercises for major muscle groups; commonly 2–4 sets of 8–12 reps, total session often ≥20 min	Hold each stretch 10–30 s, repeat 2–4 times; target ~60 s total stretching per muscle group (often <10 min/session depending on how many muscle groups are included)
Type	Walking, cycling, swimming, rhythmic activities using large muscle groups	Machines, free weights, bands, functional bodyweight exercises	Static, dynamic, and/or PNF stretching for major muscle–tendon units

Abbreviations: HRR: reserve heart rate; VO_2_R: reserve oxygen uptake; RPE: Rating of Perceived Exertion; 1RM: 1 repetition maximum.

**Table 2:** PICOS criteria for study selection.

Component	Description
Population	Adults (mean age ≥ 20 years) with elevated blood pressure (SBP 120–139 mmHg and/or DBP 70–89 mmHg) or arterial hypertension (SBP ≥ 140 mmHg and/or DBP ≥ 90 mmHg)
Intervention	Flexibility-based interventions involving stretching exercises (e.g., passive, active, static, or dynamic stretching) targeting upper limbs, lower limbs, or trunk
Comparison	Other exercise interventions or no-intervention control conditions
Outcomes	Resting blood pressure (SBP, DBP); hemodynamic parameters (MAP, HR, CO, SVR, PP, TPR); arterial stiffness indices (baPWV, CAVI, AIx); muscle flexibility
Study design	Randomized controlled trials (RCTs), randomized crossover trials, non-randomized controlled trials, and single-arm intervention studies published in English

Abbreviations: SBP: systolic blood pressure; DBP: diastolic blood pressure; MAP: mean arterial pressure; HR: heart rate; CO: cardiac output; SVR: systemic vascular resistance; PP: pulse pressure; TPR: total peripheral resistance; baPWV: brachial–ankle pulse wave velocity; CAVI: cardio–ankle vascular index; Aix: augmentation index.

**Table 3:** Characteristics of included studies.

Study	Design	Population	Intervention/Stretching Modality	Outcomes
**Wong & Figueroa (2014) **[31]	Randomized control trial.	*N* = 28 Gender: Females Age: 57 ± 1 years. ST group (*n*= 15) CON group (*n* = 14).	Chronic intervention, static unclear stretches. 18 active and 20 passive stretches; 30 s hold, 15 s rest; 3×/week for 8 weeks (~50 mins session).	Bsbp, aSBP, aDBP, aMAP, Aix, Aix@75, LFSBP, bDBP, MAP, HR, baPWV, aPWV, faPWV.
**Nishiwaki et al. (2015) **[34]	Non-randomized control trial.	*N* = 16 Gender: Males Age: 43 ± 3 years. CON group (*n* = 8) Intervention group (*n* = 8).	Chronic intervention, static unclear stretches. Static stretching of major muscles of upper/lower limb, neck, trunk muscles; 3× 20 s at end range with 30–40 s rest; 5×/week for 4 weeks (~30 mins session).	SBP, DBP, HR, handgrip strength, flexibility, baPWV CAVI.
**Boonpim et al. (2017) **[32]	Randomized control trial.	*N* = 40 Gender: Females Age: 55.00 ± 3.70 years. Control group (*n* = 20) Stretching group (*n* = 20).	Chronic intervention, static unclear stretches. Static stretching of neck, trunk, upper/lower limb muscles; 20 s hold with 40 s rest; 5×/week for 6 weeks (30 min/session).	baPWV, ankle SBP
**Silva et al. (2019) **[35]	Randomized crossover clinical trial.	*N* = 12 Gender: Males Age: 22.3 ± 2.5 years. RT-group, SS + RT group, RT + SS group, SS group	Acute intervention, Static passive stretches. RT: 3 × 10 reps at 80% of 10RM (bench press, leg extension); 2 min rest SS + RT: 2 × 30 s passive stretches + RT RT + SS: RT + 2 × 30 s passive stretches SS: 2 × 30 s passive stretches (pectorals, quadriceps); 40 s rest.	HR, DBP, SPO2, RPP, RMSSD.
**Ko et al. (2020) **[29]	Randomized clinical trial.	*N* = 40 Gender: Males & Females Age: 61.6 years. Brisk walk (*n* = 20) Stretching (*n* = 20)	Chronic intervention, static unclear stretches. 21 whole-body static stretches; 2 × 30 s, 15 s rest; 5×/week, 8 weeks (~30 min/session). Comparator: brisk walking at 50–65% of HRmax.	Nighttime DBP and MAP, sitting SBP and MAP, supine DBP and MAP.
**Yamada et al. (2022) **[38]	Single-arm prospective study.	*N* = 10 Gender: Males & Females Age: 60.1 ± 6.1 years.	Chronic intervention, static unclear stretches. Daily low-intensity 10 static stretches × 2 sets; 30 s hold; 6 months + 3 months after 1-month rest (~10 min/day).	Morning and evening SBP, morning and evening DBP.
**Higaki et al. (2022) **[33]	Randomized control trial.	*N* = 14 Gender: Females Age: 65.3 ± 6.3 years. Control group (*n* = 7) Stretching group (*n* = 7).	Chronic intervention, static unclear stretches. Lower-limb static stretching; 30 s hold, 10 s rest, 2 reps bilaterally; 2×/day, 4×/week, 4 weeks (15 min/session).	baPWV, cfPWV, HR, faPWV.
**Chen et al. (2022) **[36]	Randomized crossover clinical trial.	*N* = 13 Gender: Females Age: 72 ± 4 years. SS-group SS + FR group SS + VFR group.	Acute intervention, static unclear stretches. 3 acute crossover conditions after 5-min walk: SS (2 × 8 stretches, 30 s hold), SS + VFR, and SS + FR.	SBP, shoulder flexibility, BPP.
**Mevada et al. (2024) **[30]	Randomized clinical trial.	*N* = 98 Gender: Males & Females Age: 30.22 ± 6.32 years (Group 1), 32.20 ± 6.26 years (Group 2). Group 1 (*n* = 49) Group 2 (*n* = 49).	Acute intervention, static active stretches.	SBP, DBP, RPP, HR.
**West et al. (2024) **[37]	Randomized controlled crossover trial.	*N* = 28 Gender: Males & Females Age: 72 ± 7 years. Stretch visit (*n* = 14) Control visit (*n* = 14)	Acute intervention, static passive stretches	Central SBP, central DBP, peripheral SBP, peripheral DBP, cfPWV, MAP.
**Reyes et al. (2025) **[39]	Single-arm trial.	*N* = 24 Gender: Males & Females Age: 33 years.	Acute intervention, static active stretches.	SBP, MAP, HR.

Abbreviations: SBP: systolic blood pressure; DBP: diastolic blood pressure; RPP: Rate Pressure Product; MAP: mean arterial pressure; HR: heart rate; cfPWV: carotid–femoral pulse wave velocity; BPP: brachial blood pressure; baPWV: brachial–ankle pulse wave velocity; cfPWV: carotid–femoral pulse wave velocity; faPWV: femoral–ankle pulse wave velocity; MAP: mean arterial pressure; RMSSD: root mean square of successive differences; CAVI: cardio–ankle vascular index; AIx: augmentation index; RHI: reactive hyperemia index; ABI: ankle–brachial index; SR-group: stretching group; RT: resistance training; SS: static stretching; SS + RT: static stretching + resistance training; CON-group: control group; SS: static stretching; SS + FR: static stretching + foam roller; SS + VFR: static stretching + vibration foam roller.

## Data Availability

The original contributions presented in this study are included in the article/Supplementary Material. Further inquiries can be directed to the corresponding author.

## Supplementary Materials

The following supporting information can be downloaded at: https://www.mdpi.com/article/10.3390/jfmk11020164/s1, Table S1: Prisma checklist. Table S2: Full electronic search strategies. Table S3: Numerical findings of the included studies.

## Abbreviations

The following abbreviations are used in this manuscript:

AT	Arterial hypertension
BP	Blood pressure
SBP	Systolic blood pressure
DBP	Diastolic blood pressure
SV	Stroke volume
HR	Heart rate
RPP	Rate pressure product
MAP	Mean arterial pressure
PWV	Pulse wave velocity
baPWV	Brachial–ankle PWV
cfPWV	Carotid–femoral PWV
faPWV	Femoral–ankle PWV
RHI	Reactive hyperemia index
ABI	Brachial pressure index
CAVI	Cardio–ankle vascular index
Aix	Augmentation index
AIx@75	AIx adjusted to 75 beats min^−1^
LFSBP	Low-frequency component of SBP
RMSSD	Root mean square of the successive differences
RT	Resistance training
SPO2	Saturation
FR	Foam rolling
SS	Static stretching
VFR	Vibration foam rolling
aSBP	Aortic systolic blood pressure
aDBP	Aortic diastolic blood pressure
PP	Pulse pressure
ACSM	American college of sports medicine
PICOS	Population, Intervention, Comparison, Outcome, Study
ACC/AH	American College of Cardiology and American Heart Association criteria
ESC	European society of cardiology
