# Supplementary material for: Effects of Different Types of Stretching on Hypertension: A Systematic Review with Exploratory Meta-Analysis
Source: J Funct Morphol Kinesiol. 2026 Apr 22;11(2):164. doi: 10.3390/jfmk11020164 (PMC13108135; doi:10.3390/jfmk11020164)
Supplement: Supplementary file 1 [file jfmk-11-00164-s001.zip › Supplementary Table S2 search.pdf]

Supplementary Table S2. Full electronic search strategies

Database	Search strategy
PubMed (including MEDLINE-indexed records)	("stretching"[Title/Abstract] OR "flexibility exercises"[Title/Abstract]) AND ("hypertension"[Title/Abstract] OR "high blood pressure"[Title/Abstract] OR "pre-hypertension"[Title/Abstract])
Scopus	TITLE-ABS-KEY ("stretching" OR "flexibility exercises") AND TITLE-ABS-KEY ("hypertension" OR "high blood pressure" OR "pre-hypertension")
EBSCO (CINAHL, SPORTDiscus)	AB ("stretching" OR "flexibility exercises") AND AB ("hypertension" OR "high blood pressure" OR "pre-hypertension")

Notes: Searches were conducted from database inception to September 30, 2025. Limits applied: English language. The search strategy was adapted according to the indexing and syntax requirements of each database. Reference lists of eligible studies were also manually screened to identify additional relevant records.
