# Supplementary material for: Effects of Different Types of Stretching on Hypertension: A Systematic Review with Exploratory Meta-Analysis
Source: J Funct Morphol Kinesiol. 2026 Apr 22;11(2):164. doi: 10.3390/jfmk11020164 (PMC13108135; doi:10.3390/jfmk11020164)
Supplement: Supplementary file 1 [file jfmk-11-00164-s001.zip › Supplementary Table S3. Detailed numerical findings of the included studies.pdf]

Numerical findings of the included studies

Study	Main numerical blood pressure findings	Main numerical vascular or secondary findings
Boonpim et al. (2017)	Ankle SBP: 136.58 ± 15.79 to 123.55 ± 11.58 mmHg vs control 139.00 ± 19.11 to 140.69 ± 12.85 mmHg	baPWV: significant between-group improvement, -12% vs +6%
Chen et al. (2022)	SBP lower in SS + FR than SS and SS + VFR post-intervention; SS + FR 131.08 ± 10.29 to 132.62 ± 20.89 mmHg vs SS and SS + VFR conditions showing higher post values as reported	BPP lower in SS + FR: 55.92 ± 8.51 to 55.69 ± 16.52 vs higher post values in SS and SS + VFR; shoulder flexibility improved more in SS + VFR
Costa e Silva et al. (2019)	Post-exercise DBP lower in RT than SS: 53.93 ± 8.59 vs 76.00 ± 7.01 mmHg	HR higher in SS + RT: 90.00 ± 2.93 bpm vs RT 83.08 ± 2.32 and SS 80.09 ± 2.55; SpO2 decreased from 98.01% to 93.44% in SS; RPP higher in SS + RT than RT alone; greater RMSSD reduction when SS combined with RT
Higaki et al. (2022)	No significant differences in resting HR or BP-related measures between groups	faPWV: 1222.4 ± 167.5 to 1122.0 ± 141.1 cm/s vs control 1122.7 ± 107.7 to 1139.9 ± 77.5; no significant between-group difference in baPWV or cfPWV
Ko et al. (2021)	Sitting SBP: 146 ± 9 to 140 ± 12 vs walking 139 ± 9 to 142 ± 12; nighttime DBP: 67 ± 7 to 65 ± 10 vs 68 ± 8 to 73 ± 1; supine DBP: 85 ± 7 to 78 ± 8 vs 81 ± 7 to 82 ± 7	Favorable group × time interactions also reported for MAP outcomes
Mevada et al. (2024)	Group 1 SBP: 132.18 ± 2.21 to 125.55 ± 3.92; DBP: 86.52 ± 1.44 to 81.14 ± 2.77. Group 2 SBP: 132.86 ± 2.40 to 124.04 ± 3.23; DBP: 86.41 ± 1.35 to 79.59 ± 2.16	Significant between-group difference in SBP and DBP in favor of Group 2; no significant changes in HR or RPP
Nishiwaki et al. (2015)	No significant group × time interaction for SBP: 129 ± 5 to 133 ± 3 vs 119 ± 4 to 123 ± 3; DBP: 84 ± 4 to 87 ± 4 vs 78 ± 3 to 79 ± 2	Flexibility: 30.6 ± 5.3 to 43.9 ± 4.3 vs 31.4 ± 2.1 to 30.8 ± 2.7; baPWV: 1207 ± 28 to 1145 ± 19 vs 1204 ± 25 to 1205 ± 38; CAVI: 7.7 ± 0.2 to 7.2 ± 0.2 vs 7.6 ± 0.3 to 7.5 ± 0.3
Reyes et al. (2025)	SBP: 135.5 to 98.5 mmHg; MAP: 99.7 to 79.2 mmHg at 11 s after stretching started	HR increased from 89 to 100 bpm
West et al. (2024)	Central SBP: -3 ± 7 mmHg vs control +7 ± 6; central DBP: -2 ± 5 vs +4 ± 4. Peripheral SBP: -2 ± 8 vs +8 ± 7; peripheral DBP: -1 ± 4 vs +4 ± 4	cfPWV: 0.09 ± 0.61 m/s vs control 0.37 ± 0.68 m/s, but difference no longer significant after MAP adjustment
Wong and Figueroa (2014)	Brachial SBP: -5 ± 2 mmHg; aortic SBP: -7 ± 2; aortic DBP: -4 ± 1; aortic MAP: -8 ± 2	AIx: -6 ± 2%; AIx@75: -7 ± 2%; LFSBP: -1.62 ± 0.57 $mmHg^2$; no significant change in baPWV, aPWV, or faPWV
Yamada et al. (2022)	Morning SBP: 125 ± 10 to 125 ± 10; evening SBP: 120 ± 15 to 120 ± 9; morning DBP: 79 ± 9 to 78 ± 8; evening DBP: 74 ± 13 to 74 ± 11	No significant improvement in RHI, CAVI, or ABI over 6 months

Abbreviation: SBP: Systolic blood pressure; DBP: diastolic blood pressure; RPP: Rate Pressure Product; MAP: mean arterial pressure; HR: heart rate; cfPWV: carotid–femoral pulse wave velocity; baPWV: brachial–ankle pulse wave velocity; cfPWV: carotid–femoral pulse wave velocity; faPWV: femoral–ankle pulse wave velocity; MAP: mean arterial pressure; RMSSD: root mean square of successive differences; CAVI: cardio–ankle vascular index; AIx: augmentation index; RHI: reactive hyperemia index; ABI: ankle–brachial index; BPP: brachial blood pressure.
